# Migration intra-vésicale d’une bandelette sous urétrale type TVT: à propos d’un cas et revue de la littérature

**DOI:** 10.11604/pamj.2016.24.283.9848

**Published:** 2016-07-28

**Authors:** Adil Kallat, Oumar Diaby, Ali Valizadeh, Jean Agel, Claude Erb

**Affiliations:** 1 Service d’Urologie, Hôpital Bel-Air, CHR Metz-Thionville, France

**Keywords:** stress urinary incontinence, migration, tension free vaginal tape, conservative treatment, Stress urinary incontinence, migration, tension-free vaginal tape, conservative treatment

## Abstract

Les techniques de soutènement sous urétral par bandelette synthétique constituent le gold standard actuel pour la prise en charge de l’incontinence urinaire d’effort. Nous rapportant le cas d’une patiente de 60 ans, ayant comme antécédents une incontinence urinaire d’effort traitée par une bandelette sous urétrale type TVT (Tension free Vaginal Tape) en 2008. La cystoscopie per opératoire était normale. Six ans plus tard la patiente a présenté des infections urinaires à répétition, à Escherichia Coli, rebelles à toute antibiothérapie adaptée. Une échographie a été réalisée ayant objectivé un calcul intra vésical faisant 2.5cm de grand axe, par ailleurs le résidu post mictionnel était nul. La fibroscopie a objectivé une bandelette en intra-vésical avec une grosse calcification autour. Notre patiente a bénéficié d’un traitement conservateur qui consisté à une section endoscopique de la portion intra vésicale de la bandelette avec préservation de la portion sous urétrale, par ailleurs le calcul a été fragmenté par le lithoclaste. Sur le plan fonctionnel, notre patiente est toujours continente.

## Abstract (en)

Sub-urethral support techniques using synthetic sling are the current gold standard in the treatment of stress urinary incontinence. We report the case of a 60-year-old female patient with a personal history of stress urinary incontinence treated with suburethral TVT sling in 2008. Intraoperative cystoscopy was normal. Six years later the patient had recurrent urinary tract infections caused by Escherichia Coli, resistant to all appropriate antibiotics. The patient underwent ultrasound which objectified intravesical stone measuring 2.5 cm on the major axis; post-void residual urine volume was zero. Fibroscopy showed intravesical sling surrounded by a large calcification. Our patient underwent conservative treatment, which consisted of endoscopic section of the intravesical portion of the sling with preservation of the sub-urethral portion. The calculi was fragmented using Lithoclast. Functionally, our patientis is continent.

## Introduction

Les techniques de soutènement sous urétral par bandelette synthétique constituent le gold standard actuel pour la prise en charge de l’incontinence urinaire d’effort. Avec un recul avoisinant les dix ans, de plus en plus de patientes consultent à nouveau, souvent un autre urologue, pour divers symptômes. Certaines complications (infection, érosions diverses) peuvent nécessiter l’ablation d’une partie, voire de la totalité de la bandelette et ainsi compromettre le résultat fonctionnel acquis.

## Patient et observation

**Figure 1:**
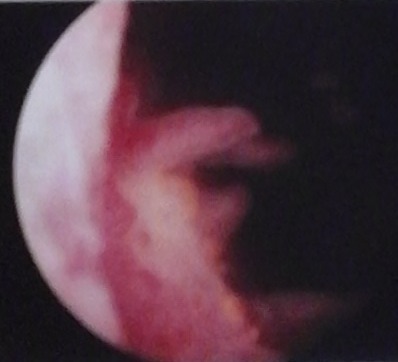
Aspect cystoscopique de l’érosion vésicale avec la bandelette en position intra vésicale

**Figure 2:**
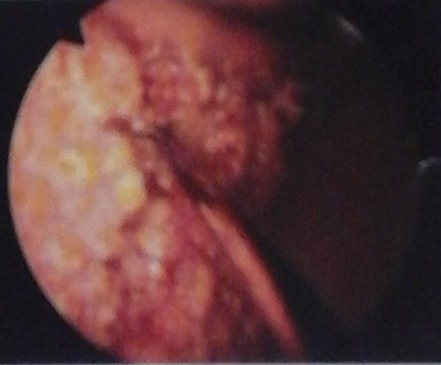
Fragmentation du calcul par le lithoclaste

Madame T.M 60 ans, ayant comme antécédents une incontinence urinaire d’effort traitée par une bandelette sous urétrale type TVT (Tension free Vaginal Tape) en 2008. La cystoscopie per opératoire était normale. Six ans plus tard la patiente a présenté des infections urinaires à répétition, à Escherichia Coli, rebelles à toute antibiothérapie adaptée. Une échographie a été réalisée ayant objectivé un calcul intra vésical faisant 2.5cm de grand axe, par ailleurs le résidu post mictionnel était nul. La fibroscopie a objectivé une bandelette en intra-vésical avec une grosse calcification autour (Figure 1). Notre patiente a bénéficié d’un traitement conservateur qui consisté à une section endoscopique de la portion intra vésicale de la bandelette avec préservation de la portion sous urétrale, par ailleurs le calcul a été fragmenté par le lithoclaste (Figure 2).

## Discussion

Les érosions vésicales représentent une complication bien connue des matériaux synthétiques. Elles peuvent correspondre à une plaie vésicale non diagnostiquée en per opératoire ou bien à une migration secondaire de la bandelette. Les symptômes amenant à suspecter l’existence d’une érosion vésicale sont des infections urinaires à répétition [1] des impériosités, une pollakiurie [2] des douleurs pelviennes ou vésicales persistantes [3] ou une hématurie [4]. Dans notre cas la migration de la bandelette a été révélée par des infections urinaires à répétition. Concernant la prise en charge thérapeutique, la voie endoscopique est envisageable en première intention. Elle peut être menée au cours d’une cystoscopie seule, Giri et al utilisent le laser holmium lors d’une fibroscopie avec un résultat per opératoire satisfaisant confirmé par une cystoscopie à trois mois [5]. Pour sectionner la bandelette le plus loin possible, il est nécessaire d’exercer une traction à l’aide d’une pince à corps étranger. Afin de faciliter la gestuelle, Lapouge et al recommandent d’associer à la voie endoscopique un second abord par le biais de deux trocarts positionnés dans la vessie. Sous contrôle optique, effectué par l’intermédiaire du cystoscope, la portion de la bandelette à sectionner est ainsi tendue au moyen d’une pince à préhension puis coupée par des ciseaux laparoscopiques [6]. Jorion et [7] Baracat et al. [8] série de six patientes proposent d’utiliser un néphroscope 24 ou 26 Fr associé à un trocart de 5 mm positionnée dans la vessie. But et al. Utilisent un résecteur à la manière d’une résection de tumeur superficielle de vessie [9].

## Conclusion

La facilité de la technique et le nombre croissant de patientes consultant pour une incontinence urinaire ne doivent toutefois pas faire oublier le respect d’une bonne indication et d’une technique opératoire rigoureuse et surtout l’importance d’un suivi régulier.
